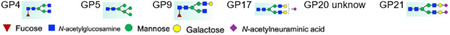# Immune and inflammatory resolution pathways through multi-omics using the AI-based Network Integration

**DOI:** 10.21203/rs.3.rs-7665795/v1

**Published:** 2025-09-23

**Authors:** Azam Yazdani, Anika Mijakovac, Franco Giulianini, Mona Alotaibi, Heike Gibson, Mohammed Ammar, Rosangela Hoshi, Richard D. Cummings, Charles N. Serhan, Gordan Lauc, Daniel Chasman, Samia Mora

**Affiliations:** Harvard Medical School; University of Zagreb; Brigham and Women’s Hospital; University of California; Brigham and Women’s Hospital; Harvard Medical School; Harvard Medical School; Harvard Medical School; University of Zagreb; Harvard Medical School; Harvard Medical School

## Abstract

Multi-omics integration faces several challenges, including heterogeneity across platforms, high dimensionality, and reduced effective sample size when all omics types are not available for every participant. We propose the AI-based Network Integration approach, which models connectivity within each omics layer as shaped by the broader biological system, including structured influences from other omics layers.

We conducted a cross-sectional multi-omics study of genetic, glycomic, and lipidomic profiles related to immune and inflammatory pathways. We integrated N-glycans of immunoglobulin G (glycans) and specialized pro-resolving lipid mediators (SPMs) to advance the understanding of immune regulation and inflammation resolution, analyzing 24 glycans and 14 SPMs. The sample size decreased from N = 456 (with glycomics) to 368 (with both lipidomics and glycomics), and further to 266 (with genetic data, lipidomics, and glycomics). Pairwise association analysis revealed 10 statistically significant glycan-SPM associations (FDR < 0.05). Using the AI-based Network Integration, we found that these associations disappeared when conditioned on two bridging molecules, 5-Hydroxyeicosapentaenoic acid (5-HEPE) SPM and a glycan peak (GP 21) corresponding to disialylation, suggesting they may be mediated or confounded by a subset of these two molecules.

Principal component analysis was applied to the previously identified associated genetic variants within each omics layer to derive polygenic factors, composite components summarizing shared genetic variation across variants. These polygenic factors were used in the Granularity Directed Acyclic Graph (G-DAG) algorithm to identify valid instrumental variables for the identification of molecular causal networks based on the principles of Mendelian randomization. This approach improved robustness and also identified polygenic molecules. Both bridging molecules, 5-HEPE and the GP21 glycan, were under polygenic control. 5-HEPE acted as a receiver (influenced by other SPMs), while GP21 acted as a broadcaster (influencing other glycans). The results suggest that variations in SPM lipidomics are linked to changes in glycosylation. These findings highlight a potential biological link between lipid mediators and glycan-mediated immune regulation and inflammation resolution.

The AI-based Network Integration approach turns noisy associations into a small, interpretable, and biologically meaningful connectivity that cannot be explained by any other component in the study.

## Introduction

A key challenge in multi-omics integration is the risk of generating findings that lack biological relevance, driven by the inherent heterogeneity across data types and measurement platforms^1,2^. Additionally, increasing dimensionality introduces analytical complexity and reduces the effective sample size, as different omics measurements are often unavailable for all samples. Evidence also suggests that connectivity within each omics layer tends to be stronger than between layers^3,4^, making the detection of meaningful cross-omics relationships especially difficult. These challenges are not adequately addressed by current multi-omics integration methods, which often rely on naive data concatenation or treat omics layers independently. To overcome these limitations, we propose the AI-based Network Integration, which is based on the premise that the structure of molecular networks within each omics layer is shaped by, and thus robust to, variability in other omics layers. This assumption is supported by systems biology, particularly in cross-sectional settings. The AI-based Network Integration first constructs molecular networks within each omics layer using all available samples (maximizing sample size) and then integrates these networks to reduce sensitivity to noise and include more samples in downstream analyses.

To infer molecular networks within each omics layer, we propose to use Bayesian networks, a class of probabilistic graphical models widely used in artificial intelligence (AI), to model both inter-omics and intra-omics relationships. In this framework, molecules are represented as nodes, and edges represent direct conditional dependencies: relationships not fully explained by any subset of other molecules in the study^5–7^. These AI-based networks and their integration with genetic information enable the identification of potential causal or regulatory relationships, offering a data-driven view of the underlying molecular architecture^8–12^. Further analysis of these causal networks allows us to characterize the roles of individual molecules, for example, those that influence many others (*broadcasters*) and those that are predominantly influenced by others (*receivers*)^13^.

To investigate cross-omics connectivity relevant to immune modulation and inflammation resolution, we integrated specialized pro-resolving mediators (SPMs), a class of bioactive lipids measured via lipidomics, and immunoglobulin G (IgG) glycans, which are glycan (sugar) structures attached to IgG antibodies measured using glycomics. IgG glycans are key modulators of IgG function, influencing its pro- or anti-inflammatory activity^14^. Alterations in IgG glycosylation have been linked to aging, inflammation, autoimmune diseases, infections, and cardiometabolic disorders^15^. In lipidomics, SPMs are lipid mediators derived primarily from omega-3 and omega-6 fatty acids^16,17^. They actively regulate the resolution phase of inflammation, promoting clearance without immunosuppression, and are increasingly studied in the context of chronic diseases such as cardiovascular disease, diabetes, and autoimmunity.

In separate studies of SPMs and IgG glycans, each has shown associations with a wide range of chronic conditions, and both are well-established regulators of immune function and inflammatory resolution. Yet, despite these shared disease links and immunomodulatory roles, no studies have examined the relationship between IgG glycans and SPMs. Here, we decided to address this unexplored question by applying the AI-based Network Integration, a novel approach we developed. This represents the first systematic effort to infer relationships between glycans and SPMs, which would lay the groundwork for a novel research direction aimed at understanding how these distinct molecular layers may interact to shape inflammatory outcomes.

## Results

**Figure 1:**
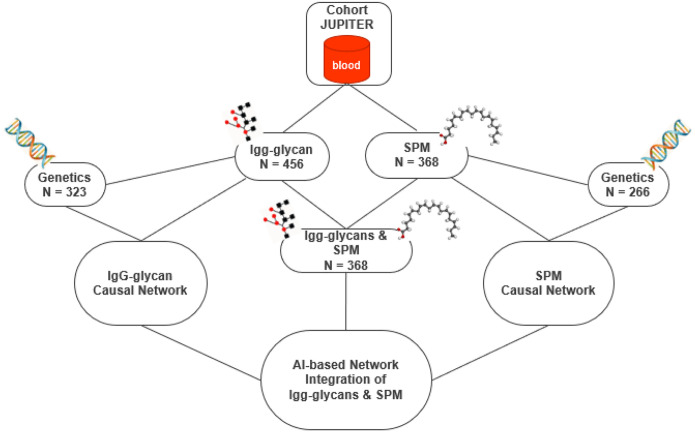
The workflow of the analysis and the sample sizes. The networks uncover the underlying relationships among molecules. Causal networks are identified based on MR principles using genetic information. Abbreviation: MR (Mendelian randomization), SPM (specialized pro-resolving mediators)

In this study, we integrated three omics layers, genetics, IgG glycans, and SPMs, with sample sizes varying across layers. We examined the connectivity both within and between IgG glycans and SPMs and used the previously identified associated genetic variants to explore potential causal relationships underlying these connections based on principles of Mendelian randomization (MR). An overview of the study design, analytical approaches, and sample sizes is presented in Fig. 1. The characteristics of participants are provided in Supplementary Table S1.

We identified 10 significant associations between 24 IgG glycans and 14 SPMs (FDR < 0.05) using 368 samples and linear regression models adjusted for age, sex, race/ethnicity, batch effect, and cardiovascular disease status (Table 1).

### The associations are ordered first by GPs and then by FDR

Among these associations, only the two molecules GP4 and 5-Hydroxyeicosapentaenoic acid (5-HEPE) are negatively associated.

**Figure 2:**
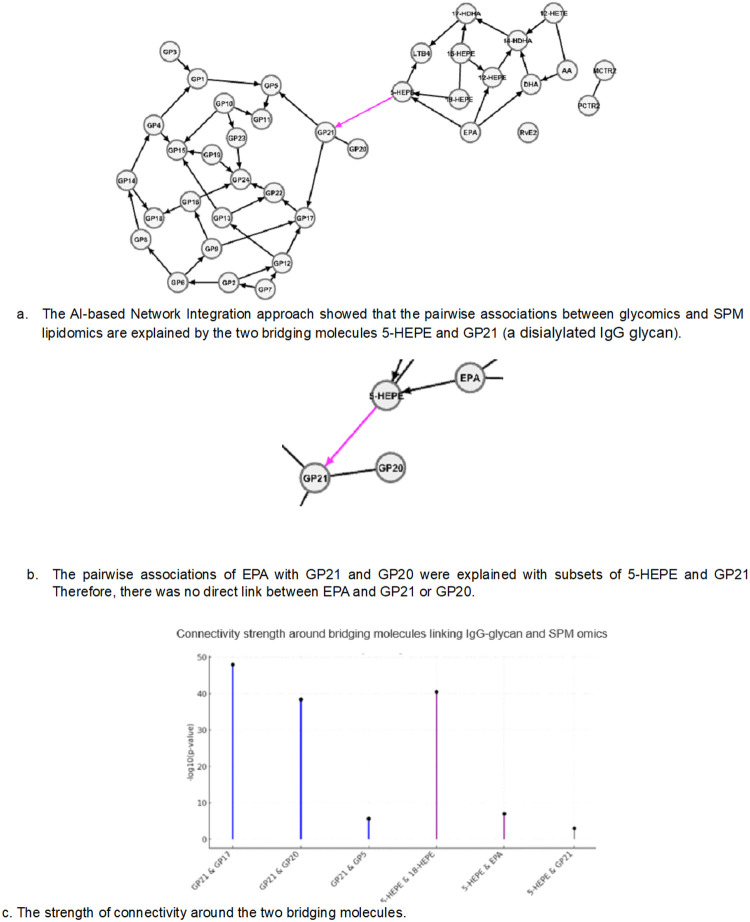
The AI-based Network Integration approach for multi-omics. **a.** The SPM causal network and IgG glycan causal network were identified at level alpha = 1e-3. Then, the networks were integrated at level alpha = 1e-2 using the AI-based Network Integration approach, and the molecules 5-HEPE and GP21 were identified as the bridging molecules. **b.** Zoom in on the network. Several pairwise associations disappeared, such as the pairwise association of EPA with GP21 and GP20, which means the associations can be explained by other sets of molecules. **c.** The connectivity strength of bridging molecules 5-HEPE and GP21 with directly connected molecules, measured by −log10(p-value). The strengths within each omics, such as GP21 with GP17, GP20, GP5, and 5-HEPE with 18-HEPE and EPA, were much larger than between the omics, 5-HEPE with GP21. Abbreviations: GP: glycan peak, SPM: specialized pro-resolving mediator, EPA: eicosapentaenoic acid

Using genetic information and the Granularity Directed Acyclic Graph (G-DAG) algorithm, we identified the IgG glycan causal network (N = 323) as well as the SPM causal network (N = 266) based on the MR principles. The directions represent the flow of information and may have causal interpretations (Methods). We identified the SPM causal network and IgG glycan causal network at level alpha = 1e-3 (Fig. 2) and assessed the stability of the networks by the Sparse Optimal Topology (SPOT) technique^9^ and the Variable Reduction Test^4^. These networks revealed the connectivity among molecules at each of these two omics, using sample size N = 456 for IgG glycans and N = 368 for SPMs. In the second step, we integrated the IgG glycan network with the SPM network using the AI-based Network Integration approach and revealed that the two layers were connected through the bridging molecules 5-HEPE and GP21 (Fig. 2).

The edges in the networks represent relationships between the two corresponding molecules that cannot be fully explained by any subset of the other molecules^7^. To illustrate how to interpret results from the AI-based Network Integration, we zoomed in on Fig. 2a around the two bridging molecules (Fig. 2b). From the 10 pairwise associations presented in Table 1, EPA was associated with both GP21 and GP20. However, when all molecules were analyzed simultaneously using the AI-based Network Integration, these associations were no longer direct. Specifically, the EPA–GP21 association was explained by 5-HEPE, and the EPA–GP20 association was explained by both GP21 and 5-HEPE. In contrast, the direct link between 5-HEPE and GP21 remained significant, indicating that this relationship could not be explained by any other subset of molecules in the study and their relationship is direct.

As expected, we observed that the connectivity within each omics is stronger than between omics (Fig. 2c).

For the identification of potential causal relationships between IgG glycans and SPM lipidomics, we used residual independence tests, and we assessed the hypothesis that the residual from regressing GP21 on 5-HEPE is dependent on 5-HEPE. However, this hypothesis was strongly rejected (p-value = 1), which is strong evidence that 5-HEPE influences GP21 (5-HEPE à GP21). The reverse regression 5-HEPE on GP21 also showed independence of the residual from GP21, but the evidence was weaker (p-value = 0.3). Although this does not allow us to infer a definitive causal direction, the results are more consistent with the hypothesis that 5-HEPE influences GP21 rather than the reverse. If this is the case, then the effect of lipids spreads in glycomics since the bridging molecule 5-HEPE is a receiver and the bridging molecule GP21 is a broadcaster. The effect of lipids spreads mostly on the IgG glycans with complex structures, including galactose and N-acetylneuraminic acid (GP17, GP22, GP24, and GP20).

#### Extracting information from the IgG glycan network

**Figure 3:**
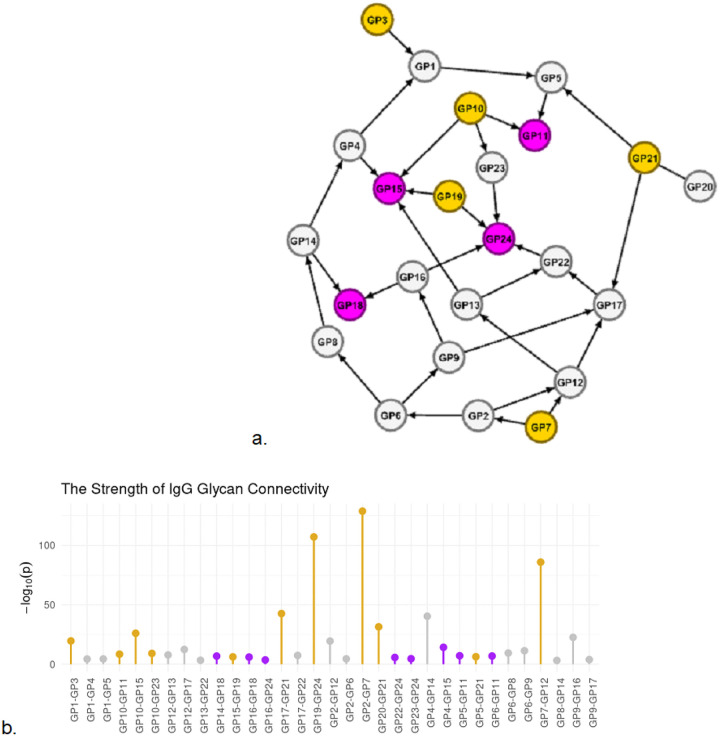
The IgG glycan Causal Network. **a.** The network connectivity of IgG glycans at level alpha = 1e-3. The directions are identified by genetic factors (not depicted in the network) based on the principles of MR. The nodes in orange are broadcasters (influence others), and the nodes in purple are receivers (influenced by others). **b.**The strength of the connections in the IgG glycan network. The highest strengths belong to broadcasters (yellow needles), while receivers have the lowest strength.

**Figure 4:**
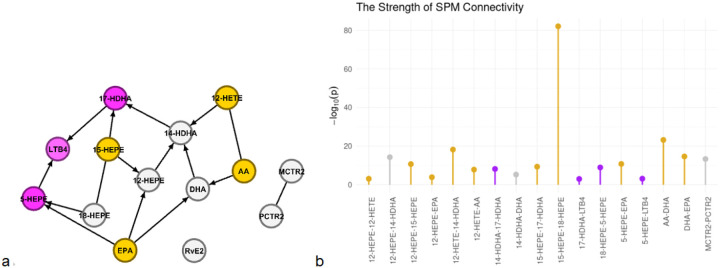
The SPM Causal Network. **a.** The network connectivity of SPM lipids at level alpha = 1e-3. The directions are identified by genetic factors (not depicted in the network) based on the principles of MR. The nodes in orange are broadcasters, and the nodes in purple are receivers. **b.** The strength of the connections in the SPM network. The highest strengths belong to the broadcaster 15-HEPE, while the absolute receiver LTB4 (only influenced by others) has the lowest strength within the network.

The IgG glycan network depicts the connectivity of IgG glycans (Figure 3a). We observed that IgG glycans belonging to the trait core fucosylation (GP24, GP18, GP15, and GP11) had the properties of receivers (influenced by other IgG glycans). Receivers are not strongly connected to the network, regardless of the number of connections. For example, GP15 and GP24 have the highest connectivity in the network (degree = 4); however, their strength (how strongly they are connected to the network) was among the lowest (Figure 3b). This is opposite for broadcasters (influence other molecules), including GP21, GP10, GP19, and GP7 related to the traits core fucosylation, disialylation, and monogalactosylation. We observed that broadcasters generally were strongly connected to the network (Figure 3b, yellow needles). This is consistent in the SPM causal network (see Figure 4b) and previous observations in the metabolomics causal network^18^, see the discussion.

The IgG glycans GP20 (with an unknown chemical structure) and GP21 (a fully sialylated, digalactosylated biantennary N-glycan) show strong statistical evidence of dependence across all tested conditioning sets of IgG glycans. The highest p-value of the conditional independence test between GP20 and GP21 is still very low (p-value = 2e-30), while this value for GP20 with other IgG glycans was significantly weaker (p-values > 0.05). This may provide meaningful clues about GP20.

#### Extracting information from the SPM network

In the SPM network, 5-HEPE and leukotriene B-4 (LTB4) had the properties of receivers, and EPA and arachidonic acid (AA) have the properties of broadcasters (Figure 4a). The highest strength belongs to the broadcaster 15-HEPE. TB4, as an absolute receiver (out-degree = 0), has the lowest strength (Figure 4b).

Maresin Conjugates in Tissue Regeneration 2 (MCTR2) and Protectin Conjugates in Tissue Regeneration 2 (PCTR2) are directly connected but independent from the SPM network at level 1e-3. Resolvin E2 (RvE2) is also independent of the network and any other SPMs.

## Methods

### Data Availability.

The study population was derived from JUPITER^19^ (Justification for the Use of Statins in Prevention: an Intervention Trial Evaluating Rosuvastatin; NCT00239681), an international, randomized, placebo-controlled trial of rosuvastatin (20mg/day) in the primary prevention of cardiovascular disease conducted among apparently healthy men and women. Here, the focus was on the participants’ baseline measurements.

Requests to access the dataset from qualified researchers trained in human subject confidentiality protocols should be sent to the Steering Committees of the parent trial.

#### Omics Profiling.

IgG glycans were measured at Genos, Inc. (Zagreb, Croatia.IgG was isolated from individual plasma samples using CIM r-Protein G LLD 0.2 mL Monolithic 96-well plate (2 μm channels; catalog no. 120.1012–2, Sartorius BIA Separations)^20^ while N-glycans were released by peptide: N-glycosidase F (PNGase F, catalog no. V4831, Promega) and labeled with a fluorescent dye, 2-aminobenzamide (catalog no. A89804, Sigma-Aldrich), for details see^21^. Prepared samples were sent to the processing laboratory, where they were stored at −20 °C until ultra-high-performance liquid chromatography analysis was performed on a Waters ACQUITY ultra-high-performance liquid chromatography analysis H-Class instrument. All chromatograms were separated in the same manner into 24 IgG glycan peaks (IgGGPs), and the amount of glycans in each peak was expressed as the percentage of the total integrated area^22^.

SPM levels were identified in plasma samples using non-targeted liquid chromatography-mass spectrometry (LC-MS) and confirmed using internal standards at Sapient Bio (San Diego, CA). The samples were spiked and SPM standardized and then purified. The SPM peaks were manually identified using exact mass-to-charge ratios (m/z), retention times, and peak shapes, complemented by MS/MS fragmentation patterns to confirm their identity. This approach ensured accurate and reliable identification of SPMs in plasma.

Genotyping was performed using the Illumina Omni 1M Quad platform (Illumina, San Diego, CA). After standard quality control procedures, 99.71% of loci yielded high-quality genotype calls, details described elsewhere^23^. All samples had successful genotyping for > 98.5% of the final single-nucleotide polymorphisms (SNPs). A total of 1,006,348 SNPs passed QC filters, of which 814,418 had a minor allele frequency >1%. These genetic variants were used for imputation via the Michigan Imputation Server^24^ using the 1000 Genomes Project pilot reference panel^25^, with Hardy–Weinberg equilibrium filtering set at p > 10^−6^.

### Analytical Approach

To integrate SPMs and IgG glycans, we focused on 368 individuals with measurements for 24 IgG glycans and 14 SPMs. Each of the 14 SPMs had no missing values or fewer than 10%, which were imputed using the mean of the observed values. An additional 13 SPMs, each with more than 50% missing data, were excluded from the analysis.

#### Linear regression.

We first performed linear regression analyses, fitting each IgG glycan to each SPM molecule. Data preprocessing for IgG glycans included total area normalization, winsorization, and standardization (mean = 0 and standard deviation = 1). The winsorization function calculated the 5^th^ and 95^th^ percentiles and replaced values outside this range with the corresponding thresholds. This method limits the impact of outliers while preserving the overall distribution of the data.

For SPMs, we applied winzorization and standardization and adjusted them for batch effects (see Supplementary for batch correction). Then, we modeled IgG glycans on SPMs. All models were adjusted for age, sex, race/ethnicity, IgG glycan batch effect, and whether they subsequently experienced a cardiovascular disease event. We selected the significant associations using the Benjamini-Hochberg method, FDR < 0.05. The results of this analysis are provided in Table 1.

#### The AI-based Network Integration approach.

We also integrated the two intermediate omics, IgG glycomics and SPMs, using the AI-based Network Integration. This approach assumes that each omics has already been influenced by existing variations in the other omics. Therefore, the molecular network structure remains unchanged despite variations in other omics layers:

$$str_{\text{omics}\;i}⊥str_{\text{omics}\;j},\;for\;\forall \;i\neq j,$$

Which means the structure (str) of omics dataset *i* is independent of the structure of omics dataset *j*, for all *i* not equal to *j* in a cross-sectional study.

We first normalized and then adjusted the molecules at each omics for batch effects, sex, age, race/ethnicity, cardiovascular disease events, and statin use. We identified the network structure using conditional dependencies and uncovered the underlying connectivity among the molecules within each omics^26^. We then integrated the networks using conditional dependencies and the Bayesian multi-trait approach^27^ to identify the connectivity between the two omics, IgG glycans and SPMs.

We identified the network structure of 24 IgG glycans using 456 samples and the network structure of 14 IgG glycans using 368 samples at level alpha = 1e-3. We considered this level using the SPOT^9^, where the Hamming distance (HD) of different networks is modeled on two consequence levels of alpha. Here, represents the significance level used in statistical tests to determine conditional dependencies between two molecules, conditioning on all sets of other molecules in the

$$\alpha_{i}=\left\{10^{-i}|i=2,3,4,5\right\}$$

and identified the optimal.

#### Causal Networks.

We used genetic variation and the G-DAG algorithm to identify the causal networks based on the principles of MR. We generated the genetic factors, assessed the MR assumptions, selected instrumental variables, and identified causal relationships between molecules^8,28^. Note that if the direction between two molecules cannot be identified, the G-DAG algorithm leaves the link undirected.

In classical MR techniques, individual genetic variants (e.g., SNPs) are used as instrumental variables that are strongly associated with exposures. However, in omics studies, we usually cannot find individual genetic variants strongly associated with each molecule in the study, and therefore, such molecules are dropped from the causal study. To overcome these limitations of classical MR, we previously introduced an approach for generating polygenetic factors^8^, so that each factor captures variation in multiple single genetic variants and may show a stronger association with molecules in the study. This approach is suitable for omics studies and causal inference. In previous studies, we applied both principal component analysis^29^ and multiple correspondence analysis^4^ to generate polygenic factors; in the present study, we used principal component analysis.

Generating polygenic factors prevents spurious estimates and inflated Type 1 errors when using too many genetic variants in the analysis or highly sensitive estimates due to ignoring most data and using a few genetic variants. In addition, the results of the MR approach using polygenic factors are more robust to seemingly arbitrary choices in the variable selection step^28^.

For generating polygenic factors, we took the steps below for each omics separately (for details see Supplementary, Sections 4–5):

1. Extracted genetic variants associated with each omics layer (IgG glycans and SPMs) from the GWAS Catalog (https://www.ebi.ac.uk/gwas/). These variants were then identified in the imputed genotype data from the JUPITER cohort. In total, we retrieved 455 genetic variants for IgG glycans and 750 for SPMs.
2. Applied linear regression analysis, fitting each molecule on each variant and adjusting models for 5 first five principal components (PC) for population stratification and covariates sex, age, and race/ethnicity. We selected variants at p-value < 0.05 at this stage; in total, 84 variants were associated with SPMs and 116 with IgG glycans.
3. Generated polygenic factors by applying principal component analysis on the selected genetic variants in step 2 at each omics to extract variations from the variants.
4. Applied the G-DAG algorithm to the polygenic factors and molecules in each omics. The polygenic factors with strong association with molecules and the lack of pleiotropy were used as instrumental variables for causal inference based on the MR principles.
5. Evaluated the stability of causal networks using the Variable Reduction Test^4^. In the networks, we considered molecules/nodes with the role of receivers. The property of such nodes is arrows pointing in, with no arrows pointing out. This means the corresponding molecules do not influence any other molecules. Therefore, if we remove the corresponding variables from the set of variables and then identify a network of the subset, we expect the directions among the variables in the subset to stay the same as before. To apply this test, we permuted one receiver at a time (Supplementary, Section 6).

For the potential causal relationship between the two omics, we used residual independence tests, and we assessed the hypothesis that the residuals from regressing GP21 on 5-HEPE are dependent on 5-HEPE. However, this hypothesis was strongly rejected (p-value = 1). The reverse, regressing 5-HEPE on GP21, also showed independence, but the evidence for rejecting the hypothesis was weaker (p-value = 0.3).

## Discussion

In this study, we addressed key challenges in multi-omics integration, such as data heterogeneity, limited sample overlap, and analytical complexity, by introducing the AI-based Network Integration approach in the Causal Framework. This framework leveraged molecular connectivity and causal inference and enabled integration across omics layers without unnecessarily increasing dimensionality, while maximizing the use of available samples, even when samples only partially overlapped. As a result, we uncovered biological insights related to immune modulation and inflammation resolution. Specifically, we found that the two molecules 5-HEPE (an omega-3 EPA-derived lipid mediator) and GP21 (a disialylated IgG glycan) were key bridging molecules between lipidomics and glycomics.

The causal network inference revealed 5-HEPE as a receiver and GP21 as a broadcaster, suggesting lipid changes may influence glycan profiles and IgG function, supporting a lipid-to-glycan signaling axis relevant to inflammation resolution. In addition, the current study found evidence that lipids may influence more complex IgG glycans, i.e., those glycans with galactose and sialic acid structures. Interestingly, both 5-HEPE and complex galactosylated and sialylated IgG glycans are generally considered protective in the context of inflammation^15^. The loss of these glycans has been associated with various cardiometabolic and autoimmune conditions, diseases in which SPMs, such as 5-HEPE, have also been shown to play a protective role^30^. In this study, we found that IgG glycans containing galactose and sialic acid were positively associated with multiple SPMs. Conversely, GP4, an agalactosylated, core-fucosylated glycan elevated in both cardiometabolic and autoimmune diseases, exhibited a negative association with 5-HEPE. These findings demonstrate the potential of the AI-based Network Integration approach to generate results with high biological interpretability.

This work also uncovered the influence pattern within the glycan network. IgG glycan pairs showing the strongest connectivity also occupied sequential positions in the biosynthetic cascade of glycosylation. For example, GP2 (agalactosylated IgG glycan without core fucose) is converted to GP7 (monogalactosylated IgG glycan without core fucose) in a single step by the action of a galactosyl-transferase. These known relationships illustrate how strong connectivity relates to underlying biology^31^. In addition, broadcasters seem to encompass less complex IgG glycans, and receivers, more complex and biologically less complex glycans serve as substrates for the more complex ones.

The finding that IgG glycans with the strongest connectivity correspond to successive steps in the glycan biosynthesis pathway illustrates how strong connectivity could reflect underlying biology even within the omics layer. Considering that the biosynthetic steps of many molecules remain incompletely characterized, the ability of the AI-based Network Integration to generate testable hypotheses both within and between the omics layers is a powerful tool. It can be used to discover possible biological relationships and guide future experimental validation, as demonstrated in one of our previous studies that used network inference to propose a correction of a wrongly mapped IgG biosynthesis pathway, which was subsequently experimentally validated^31^.

The 5-HEPE is the EPA product of the leukocyte 5-lipoxygenase. It can be further converted to RvE2 and Lipoxin A5^32^. Both 5-HEPE and EPA are known to regulate IgG production^33^. Some of the connections revealed here may diverge from classical biosynthetic pathways, such as those described for SPMs^34^. Indirect connections identified in data-driven networks may reflect underlying biochemical interactions or regulatory relationships between molecules. Similarly, direct connections could also represent such relationships, even in the absence of experimentally validated enzymatic steps. Importantly, neither biosynthetic pathways (e.g., SPM and glycan biosynthesis as defined in prior literature) nor data-driven networks should be considered definitive. Both serve as provisional frameworks that will evolve with new experimental findings and methodological advances. The study here in the causal framework will benefit from a larger sample size. The results were more consistent with the hypothesis that 5-HEPE influences GP21 rather than the reverse. However, a definitive causal inference between glycomics and lipidomics could not yet be established.

The AI-based Network Integration approach applied here enhances the interpretability of the findings by revealing potential mechanistic relationships among molecular components. This approach advances the field by overcoming several key challenges reviewed earlier and by uncovering novel cross-omics connections. These findings represent an important step forward and open new avenues for research. This study introduced the novel AI-based Network Integration in the causal framework for cross-sectional multi-omics data that differs fundamentally from traditional strategies, which typically concatenate omics data or analyze each omic type in isolation. Importantly, it lays the groundwork for causal inference across omics layers. Moreover, the framework is readily extensible to longitudinal and spatial data, making it a scalable and versatile tool for future systems biology research.

By utilizing this newly developed AI-based methodology, this study is the first, to our knowledge, to infer relationships between IgG glycans and SPMs through the integration of genetic, glycomic, and lipidomic data. The discovery of 5-HEPE and GP21 as bridging molecules between lipidomics and glycomics gives a new insight into immune modulation and inflammation. By establishing a foundational framework for identifying potential causal links between IgG glycans and SPMs, we opened a new, unexplored avenue of research that could ultimately inform targeted strategies to curb chronic inflammation, a defining feature of most non-communicable diseases.

## Figures and Tables

**Table 1:** The significant associations between IgG glycan peaks (GP) and specialized pro-resolving mediators (SPMs) (pairwise associations)

	Effect size	SE	p-value	FDR
GP21, 5.HEPE	0.32	0.05	4.4 e-10	1.5 e-7
GP21, EPA	0.30	0.05	2.2 e-8	3.6 e-6
GP21,18.HEPE	0.19	0.05	2.1 e-4	1.2 e-2
GP20, EPA	0.28	0.05	1.3 e-7	1.4 e-5
GP20, 5.HEPE	0.24	0.05	1.5 e-6	1.2 e-4
GP17, EPA	0.19	0.05	1.1 e-3	3.6 e-2
GP17, 5.HEPE	0.20	0.05	2.2 e-4	1.2 e-2
GP9, EPA	0.20	0.05	2.6 e-4	1.3 e-2
GP5, EPA	0.19	0.05	7.8 e-4	3.3 e-2
GP4, 5.HEPE	−0.17	0.05	1.1 e-3	3.6 e-2

GP: glycan peak; HEPE: Hydroxyeicosapentaenoic acid; EPA: eicosapentaenoic acid;

SPM: specialized pro-resolving mediator, SE: standard error

Traits: GP4 & GP9 (Asialylation), GP5 (−), GP17 & GP20 (Monosialylation), GP21 (Disialylation)

Glycan structure: